# Fatal hemorrhagic varicella in a patient with abdominal pain: a case report

**DOI:** 10.1186/s12879-019-4716-6

**Published:** 2020-01-17

**Authors:** Wei Zhang, Qiao-ling Ruan, Fang Yan, Yue-kai Hu

**Affiliations:** 1 Department of Infectious Diseases, Huashan Hospital, Fudan University, 12 Wulumuqi Zhong Road, Shanghai, 200040 China; 2 Department of Dermatology, Huashan Hospital, Fudan University, 12 Wulumuqi Zhong Road, Shanghai, 200040 China

**Keywords:** Hemorrhagic varicella, Abdominal pain, Next-generation sequencing, Varicella-zoster virus, Multi-organ failure

## Abstract

**Background:**

Varicella is normally a self-limited childhood disease caused by varicella-zoster virus infection. However, it sometimes causes severe diseases, especially in immunocompromised individuals. We report a case of severe varicella in a young woman.

**Case presentation:**

A 19-year-old woman presented to the emergency department with abdominal pain and a rash after taking methylprednisolone for 2 weeks for systemic lupus erythematosis. The laboratory data showed leukocytosis, thrombocytopenia, an elevated level of the liver transaminases and disseminated intravascular coagulation. Computed tomography of the abdomen revealed multiple air-fluid levels in the intestines. Hemorrhagic varicella was considered and antiviral therapy as well as immunoglobin were applied. Her condition deteriorated and she eventually died due to multi-organ failure and refractory shock. Next-generation sequencing performed on fluid from an unroofed vesicle confirmed the diagnosis of varicella.

**Conclusion:**

In its severe form, VZV infection can be fatal, especially in immunocompromised patients. Hemorrhagic varicella can be misdiagnosed by clinicians because of unfamiliar with the disease, although it is associated with a high mortality rate. In patients with suspected hemorrhagic varicella infection, antiviral therapies along with supportive treatment need to be initiated as soon as possible in order to minimize the case fatality rate.

## Background

Varicella is normally a self-limited disease caused by varicella-zoster virus(VZV) infection. However, it sometimes causes severe diseases, especially in immunocompromised individuals such as those are taking chemotherapy or steroid therapy, and those who have had a renal transplant or a bone marrow transplant [1–4]. Few immunocompetent hosts were also involved [5, 6].

We describe a case of atypical varicella in a woman who presented with abdominal pain and a rash after being treated with methylprednisolone.

## Case presentation

A 19-year-old Chinese woman was admitted to the emergency department of our hospital because of a three-day absence of defecation and a one-day history of abdominal pain. Two weeks previously, she had been discharged from the Department of Dermatology with a diagnosis of systemic lupus erythematosus(SLE). She was being treated with methylprednisolone at a dose of 24 mg per day.

**Fig. 1:**
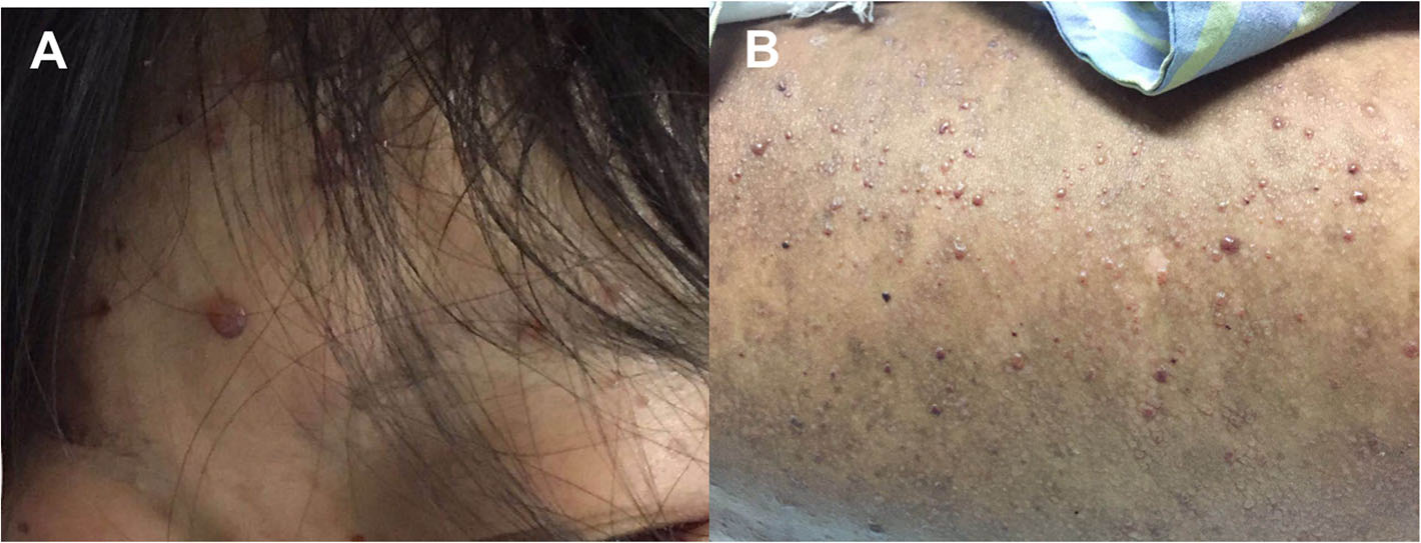
The forehead**(a)** and the trunk**(b)** of the patient in the case study. The picture shows a diffuse brown pigmentation on the skin of the patient on admission, with a superimposed vesiculopapular rash

**Fig. 2:**
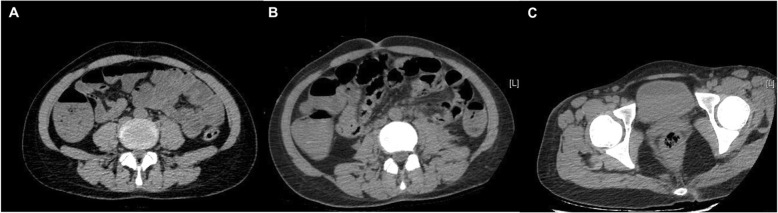
CT of the abdomen. **a**. The CT scan shows multiple air-fluid levels in the bowels suggestive of bowel obstruction on admission. **b**. The CT scan shows extensive free gases and fluids in the colon four hours after admission. **c**. The CT scan shows a high density mass in the bladder suggestive of cystorrhagia four hours after admission

On examination, there was a diffuse brown pigmentation on the skin, with vesiculopapular rash on the forehead and over the trunk (Fig. 1). There was tender on palpation of the epigastric area, without rebound or guarding. The intestinal sounds were diminished. No palpable abdominal mass or hepatosplenomegaly was observed. The emergency computed tomography (CT) of the abdomen revealed multiple air-fluid levels in the intestines. Intestinal obstruction was suspected (Fig. 2a) and an emergency surgery was arranged. Before surgery, laboratory testing revealed leukocytosis, thrombocytopenia and elevated liver transaminases. In addition, prolonged prothrombin time and partial thromboplastin time, decreased fibrinogen and increased D-dimer level indicated disseminated intravascular coagulation (DIC)(Table 1). The surgery was therefore cancelled, and plasma and prothrombin complex were administered. Gastrointestinal decompression was implemented and then plenty of blood was drained out. Four hours after admission, she suffered from an epileptic seizure for five minutes. Cranial CT ruled out intracranial hemorrhage, but a repeated abdominal CT showed an increased amount of free gases and fluids in the colon (Fig. 2b) and cystorrhagia was suspected (Fig. 2c). A urinary catheter was inserted and hematuria was observed. The patient was transferred to the intensive care unit to monitor vital signs more closely. Hemorrhagic varicella was considered and antiviral therapy of ganciclovir at 2.5 mg/kg/day as well as immunoglobin at 20 g/day were applied. However, six hours after admission, her blood pressure stared to decrease and norepinephrine was administered. Twelve hours after admission, repeated laboratory testing showed non-corrected leukocytosis and thrombocytopenia, elevated liver transaminases and a considerably prolonged prothrombin time and partial thromboplastin time. In addition, anemia and elevated creatinine was also revealed (Table 1). Then, blood oxygen saturation started to decrease and a trachea cannula was carried out. Eighteen hours after admission, the patient died due to multi-organ failure and refractory shock. Next-generation sequencing (NGS) performed on fluid from an unroofed vesicle confirmed the diagnosis of varicella (the reads of human herpes virus 3 is 32,486, accounting for 99.90%).

**Table 1:** Selected laboratory data of the patient

Variable	Reference range	20 days before admission	On admission	12 h post admission
White blood cell count (× 10^9^/L)	3.5–9.5	7.78	35.9	24.02
Neutrophil (%)	40.0–75.0	81.8	80	85
Hemoglobin(g/L)	130–175	113	143	83
Hematocrit (%)	40–50	34.9	42.4	24.4
Platelet(×10^9^/L)	125–350	417	50	56
Alanine aminotransferase (U/L)	9–50	18	872	814
Aspartate aminotransferase (U/L)	15–40	19	2061	1885
Urea nitrogen (mmol/L)	2.5–7.0	2.3	8	13.2
Creatinine (μmol/L)	50–130	36	65	146
International normalized ratio (INR)	0.92–1.15	0.88	3.01	4.18
Prothrombin time (sec)	10.7–13.1	10.4	33.6	45.3
Partial thromboplastin time (sec)	20.3–32.3	24.1	82.7	131.7
Fibrinogen (g/L)	1.8–3.5	1.9	0.5	NA^a^
D-dimer (FEUmg/L)	≤0.55	0.84	10.25	NA^a^

^a^*NA* Not available because of interfering substance

## Discussion and conclusions

In its severe form, VZV infection can be fatal, especially in immunocompromised patients. Visceral disseminated disease is associated with a high mortality rate of 46–55% [7]. In normal varicella infection, as Kole AK et al. reported, hemorrhage vesicle is a relative unusual complication occurred in 3.3% of the patients [8]. However, it’s more common in visceral disseminated varicella. In a report of 38 cases of visceral disseminated varicella, rash was the presenting complaint in 89% of the cases [9]. However, some patients have no skin involvement, which probably leads to misdiagnosis and poses diagnostic and therapeutic challenges, especially when some other disease manifestations exist [7, 10]. Despite skin involvement, the symptoms of disseminated varicella also include multiple hemorrhage (including intracranial hemorrhage, hemorrhagic gastritis, hemorrhagic pulmonary edema, splenic rupture, adrenal hemorrhage, cystorrhagia and hyphema), encephalitis, pneumonia and abdominal pain [11–20].

Intense abdominal pain is often an early symptom of dissemination, which reveals that multi-system organs are involved, such as the stomach, intestines and spleen (which may lead to hemorrhagic gastritis, intestinal obstruction and splenic rupture) [12, 14, 21]. Abdominal pain usually presents earlier before the appearance of the rash with a mean interval of 6.5 days [9, 20, 22]. Visceral VZV infection often presents as epigastric abdominal pain, occasionally involving the right upper quadrant or radiating to the back [23].The patient in our case began with abdominal pain and was initially diagnosed with intestinal obstruction, which led to schedule an emergency laparotomy. Thrombocytopenia and DIC was observed, which may have lead to the occurrence of gastrorrhagia, cystorrhagia and finally hemorrhagic shock. A review of 270 patients with varicella infection found that thrombocytopenia was very common, with 30% of patients having a platelet count < 150 × 10^9^/L [24]. Although our patient did not have purpura, purpura is often a sign of thrombocytopenia. The estimated incidence of thrombocytopenic purpura as a complication of varicella infection is approximately 1:25,000 [25]. In addition, varicella is frequently accompanied by some degree of DIC, particularly in immunocompromised patients [26].

VZV is usually confirmed by polymerase chain reaction (PCR) amplified VZV DNA and enzyme-linked immunosorbent assay (ELISA) detection of anti-VZV antibody [27]. In our case, we applied a novel method, referred to as next-generation sequencing (NGS). As a complementary method to conventional methods of diagnosis such as viral isolation in cell culture, viral detection by molecular testing and genetic analysis by cycle sequencing, NGS provides a powerful tool to address the challenges of viral infections, which has been applied to a metagenomics-based strategy for rapid and accurate discovery and characterization of new viruses and detection of unexpected viral pathogens in clinical specimens [28].

The patient with SLE in our case was just discharged from the Department of Dermatology, where she had many opportunities for exposure to patients with herpes zoster or varicella. And the patient had never been vaccinated against varicella before. Vaccination of close contacts should be considered for immunocompetent individuals [29]. However, for immunocompromised individuals, VZV vaccination is contraindicated and can be fatal because the vaccine contains live attenuated virus. In case of exposure, medical care should be sought immediately as varicella-zoster immune globulin (VariZig) may be effective in reducing disease severity and should be administered as soon as possible after exposure [30]. However, VariZig is not available in China up to now because it has not been licensed. As clinicians, once virus infection of immunocompromised patients is observed or considered, antiviral therapies should been initiated along with supportive treatment as soon as possible so that the patients can have a slim chance of survival.

## Data Availability

All data contained within the article.

## Abbreviations

CT: Computed tomography
DIC: Disseminated intravascular coagulation
ELISA: Enzyme-linked immunosorbent assay
NGS: Next-generation sequencing
PCR: Polymerase chain reaction
SLE: Systemic lupus erythematosus
VariZig: Varicella-zoster immune globulin
VZV: Varicella-zoster virus
